# Analyses of apoptotic regulators CASP9 and DFFA at 1P36.2, reveal rare allele variants in human neuroblastoma tumours

**DOI:** 10.1038/sj.bjc.6600111

**Published:** 2002-02-12

**Authors:** F Abel, R-M Sjöberg, K Ejeskär, C Krona, T Martinsson

**Affiliations:** Department of Clinical Genetics, Gothenburg University, Sahlgrenska University Hospital/East, S-416 85 Gothenburg, Sweden

**Keywords:** 1p-deletion, neuroblastoma, neuroectodermal, Apaf-3, DFF, ICAD

## Abstract

The genes encoding Caspase-9 and DFF45 have both recently been mapped to chromosome region 1p36.2, that is a region alleged to involve one or several tumour suppressor genes in neuroblastoma tumours. This study presents an update contig of the ‘Smallest Region of Overlap of deletions’ in Scandinavian neuroblastoma tumours and suggests that DFF45 is localized in the region. The genomic organization of the human DFF45 gene, deduced by in-silico comparisons of DNA sequences, is described for the first time in this paper. In the present study 44 primary tumours were screened for mutation by analysis of the genomic sequences of the genes. In two out of the 44 tumours this detected in the DFFA gene one rare allele variant that caused a non-polar to a polar amino acid exchange in a preserved hydrophobic patch of DFF45. One case was hemizygous due to deletion of the more common allele of this polymorphism. Out of 194 normal control alleles only one was found to carry this variant allele, so in respect of it, no healthy control individual out of 97 was homozygous. Moreover, our RT–PCR expression studies showed that DFF45 is preferably expressed in low-stage neuroblastoma tumours and to a lesser degree in high-stage neuroblastomas. We conclude that although coding mutations of Caspase-9 and DFF45 are infrequent in neuroblastoma tumours, our discovery of a rare allele in two neuroblastoma cases should be taken to warrant further studies of the role of DFF45 in neuroblastoma genetics.

*British Journal of Cancer* (2002) **86**, 596–604. DOI: 10.1038/sj/bjc/6600111 www.bjcancer.com

© 2002 Cancer Research UK

## 

Neuroblastoma, a paediatric tumour originating in the neural crest cells, is a heterogeneous disease with tumour progression or spontaneous regression dependent on anatomic stage and age at diagnosis. Of a group of chromosomal aberrations proven to correlate with the prognosis of neuroblastoma, the most common are deletion in the short arm of 1p (1p36.2-3) and amplification of the proto-oncogene MYCN on 2p24.1 (reviewed in Brodeur, 1990). More recently, gain of parts of the long arm of chromosome 17 (17q gain) has also been shown to have prognostic value (Abel *et al*, 1999; Bown *et al*, 1999).

Apoptosis or programmed cell death (PCD) is responsible for the maintenance of homeostasis in tissues as well as in embryonic development. Called caspases; the key apoptotic effectors comprise a family of cysteine proteases that requires cleavage after a specific internal Asp residue for activation (Li *et al*, 1997; Srinivasula *et al*, 1998). Caspase-9, a 45-kDa protein (also called ‘Apoptotic protease activating factor-3’, Apaf-3, ICE-LAP-6, Mch6), has been shown to be a critical and furthest upstream member of the mitochondrial-mediated apoptotic protease cascade (Li *et al*, 1997; Kuida *et al*, 1998; Srinivasula *et al*, 1998). Activated by Apaf-1-mediated oligomerization, Procaspase-9 in turn activates by proteolytic cleavage downstream caspases such as caspase-3, -6 and -7 (Li *et al*, 1997; Srinivasula *et al*, 1998). Caspase-3 in turn cleaves a selected group of substrates that cause the morphological and biochemical changes that characterize apoptotic cell death. One of the caspase-3 downstream substrate proteins is DFF (DNA Fragmentation Factor; Liu *et al*, 1997; Enari *et al*, 1998; Halenbeck *et al*, 1998); it is a heterodimeric protein composed of one 40- and one 45-kDa subunit (Liu *et al*, 1997). DFF40 (synonymous names CPAN ‘Caspase-Activated Nuclease’/CAD ‘Caspase-Activated Dnase’) is a DNase that triggers both DNA fragmentation and chromatin condensation (Liu *et al*, 1998). DFF45 (also called ICAD ‘Inhibition of CAD’) is an inhibitor of DFF40, that also functions as a chaperone for native DFF40 (Gu *et al*, 1999; Sakahira *et al*, 2000).

Dysregulation of apoptosis is likely to be instrumental in the development and/or progression of childhood tumour neuroblastoma. Apoptotic factors correlate in several respects with prognosis of neuroblastoma. Bcl-2, an apoptotic repressor located as a transmembrane protein in the mitochondria, endoplasmatic reticulum and nuclei, has been reported to provide prognostic information in some neuroblastoma (Ikeda *et al*, 1995; Mejia *et al*, 1998). Moreover, Ikeda *et al* (1997) reported that caspase-1 is preferentially expressed in neuroblastoma with favourable prognosis. Also, Caspase-8 is poorly expressed in neuroblastoma tumours due to deletion or inactivation by methylation (Teitz *et al*, 2000). Recently, structure and mutation analysis of the gene encoding DFF40, DFFB, was performed in primary neuroblastoma tumours and cell-lines (Judson *et al*, 2000). However, DFFB could not be shown to be imprinted or to contain any somatic mutations in tumour samples. Gene-targeting studies of CASP9 have shown that it is important during brain development. A majority of CASP9 knockout mice has been shown to die perinatally with a markedly enlarged and malformed cerebrum caused by reduced apoptosis (Hakem *et al*, 1998; Kuida *et al*, 1998). No DFF45/ICAD-deficient mouse has shown any developmental abnormality, but some cell types have shown reduced DNA fragmentation and DNA condensation and are partially resistant to undergo apoptosis (Zhang *et al*, 1998, 1999).

Both the CASP9 (OMIM number 602234) and the DFFA (OMIM number 601882) genes have recently been localized to 1p36.2 (Leek *et al*, 1997; Hadano *et al*, 1999), a region commonly deleted in neuroblastoma tumours. DFFA is in fact localized in the 1p-deleted SRO (smallest region of overlap) at 1p36.2-3 defined by our group, and according to Ohira *et al* (2000) within a homozygously deleted region in a neuroblastoma cell line. CASP9 is localized at 1p36.21, approximately 4 Mb proximal to our SRO-region, but still in the region commonly deleted by neuroblastoma tumours. In the present study, we determined the organization of the human gene encoding DFF45, DFFA, by in-silico cloning. Based on the hypothesis that neuroblastomas are tumours in which mutated cells have abnormalities in their programmed cell death and the fact that CASP9 and DFFA are localized in the neuroblastoma tumour-suppressor hot-spot region (1p36.2-3), the purpose of the present study was to investigate whether mutated CASP9 or DFFA could be causative in the development of neuroblastoma tumours in childhood.

## MATERIALS AND METHODS

### Patients and controls

DNA was extracted from frozen (−70°C) tumour samples obtained from 44 Scandinavian patients with neuroblastoma of all different stages ( Table 1

**Table 1:**
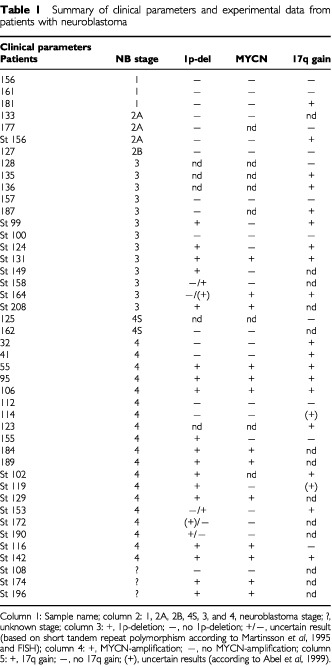
Summary of clinical parameters and experimental data from patients with neuroblastoma

). Three were stage 1, three were stage 2A, one was stage 2B, 13 were stage 3, two were stage 4S, 17 were stage 4, and five were of unknown stages. Clinical data were derived from Martinsson *et al* (1995) and from unpublished data. The neuroblastomas were staged according to the International Neuroblastoma Staging System criteria (INSS; Brodeur *et al*, 1993). DNA was also extracted from EDTA-blood obtained from 97 normal control individuals from western Sweden. If a DNA variation was found in a patient, in general, 47 normal controls were screened in order to determine the normal allele frequency of this variant (i.e. 94 alleles). If the polymorphism could not be found in this set of 47, another set of 50 normal controls were screened (i.e. in total 194 alleles).

### Mapping and BAC (bacterial artificial chromosome)-contig construction

**Figure 1:**
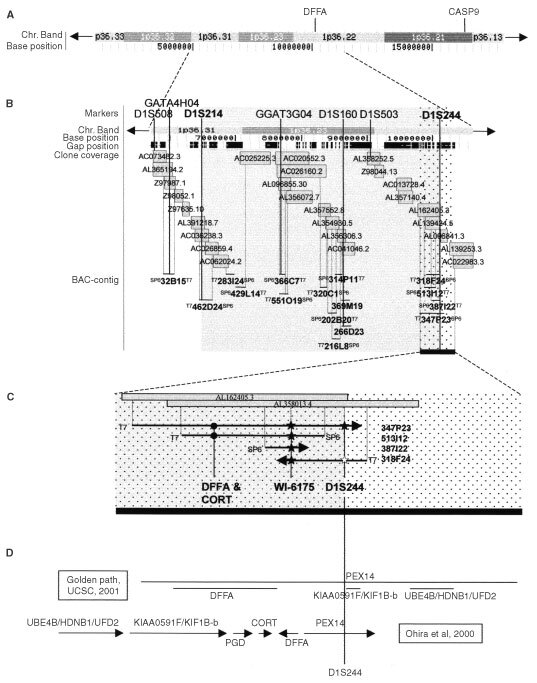
Schematic representation of the localization of CASP9 and DFFA based on alignment-search (‘BLAT-research’) data from ‘Golden path’ at UCSC (http://genome.ucsc.edu). (**A**) Chromosome band 1p36.13-33 corresponding to approximately 15 Mb, with base position according to UCSC. CASP9 is localized to 1p36.21, and DFFA is localized to 1p36.22. (**B**) Clone coverage at chromosome band 1p36.22-31 corresponding to approximately 5 Mb. Gap positions in the UCSC-contig are shown at the top. Grey boxes represent clones from ‘Golden path’. Black horizontal bars represent BAC-clones found by our group by PCR-based screening of a BAC-library (Research Genetics). BACs are located according to alignment of BAC-ends to UCSC-clones (dotted vertical lines), and their content of polymorphic markers (black vertical lines). The shaded area represents the SRO of deletions defined earlier by our group (Ejeskar *et al*, 2001). The dotted area and the thick black bar in the right corner represent the homozygously deleted region found by Ohira *et al* (2000) that partially overlaps the shaded SRO-region. (**C**) Enlarged view of the homozygously deleted region found by Ohira *et al* (2000). Grey boxes represent two clones from ‘Golden path’ (UCSC). BACs are ordered according to alignment of BAC-ends and markers to UCSC-clones (dotted vertical lines). T7 and SP6 display the two BAC-ends sequenced with the T7- and SP6-universal primers. Arrowheads represent BAC-ends ending up in UCSC gap positions, or BAC-ends with no available sequence for alignment-search (see text for details). Markers D1S244, and WI-6175 (*) and the genes DFFA and CORT (•) are mapped to the BACs by PCR assay (black vertical lines). Filled and white figures represent positive and negative PCR-results, respectively. (**D**) Schematic representation of the order of known genes believed to reside in the region by marker D1S244, proposed by ‘Golden path’ at UCSC (http://genome.ucsc.edu) and Ohira *et al* (2000).

CASP9 and DFFA were mapped by running an alignment search (BLAT-search) of each of the complete coding sequences (NM_001229 and NM_001229) on ‘Golden path’ at UCSC (http://genome.ucsc.edu; Figure 1A

). BACs were found by screening a BAC-library (Research Genetics, Huntsville, AL, USA). Some of the 16 BACs in the present study are from earlier published results (Ejeskar *et al*, 2001); while some are new (Figure 1B). BAC-ends were sequenced using Big Dye Terminator chemistry (Applied Biosystems, Foster City, CA, USA) and the universal T7- and SP6-primers. The BAC-contig was constructed by running alignment searches of seven markers (D1S508, GATA4H04, D1S214, GGAT3G03, D1S160, D1S503 and D1S244) and all available BAC-end sequences on ‘Golden path’ at UCSC. The markers and BACs were then aligned to UCSC-clones (Figure 1B). Marker D1S244 and the DFFA (exons 1 and 5) and the CORT genes, were fine-mapped by PCR-assay (data not shown) against four BACs (318-F24, 513-I12, 347-P23 and 387-I22; Figure 1C). All PCR's were run with positive and negative controls, and against two different colonies from the same BAC.

### PCR-amplification

**Figure 2:**
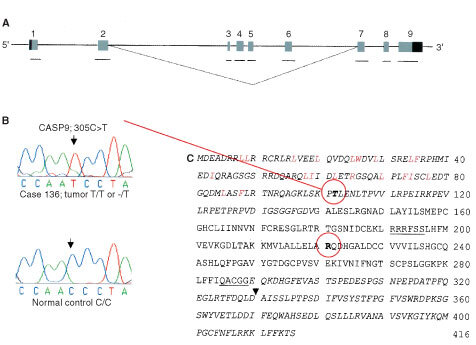
Schematic representation of the CASP9 gene. (**A**) Exon-intron organization of the gene corresponding to approximately 5 kb from (Hadano *et al*, 1999). Exons are numbered from 1–9 in the 5′-3′ direction. Light shaded boxes represent the coding regions of each exon. Dark shaded boxes represent the 5′- and 3′-untranslated flanking regions (UTR's). The black bars represent the nine amplified fragments. The dotted line represents the alternatively spliced CASP9S. (**B**) Rare polymorphism detected in CASP9. The 305C>T polymorphism located in CASP9 exon 2. Upper panel: Amplified tumour-DNA from case 136, homozygous T/T or hemizygous -/T. Lower panel: Amplified normal-DNA from control Q309, homozygous C/C. (**C**) The amino acid sequence of caspase-9 and caspase-9S (GenBank accession numbers: NM_001229 and AF110376 respectively). The caspase-9S sequence is represented in italics. The consensus Akt phosphorylation motif RRRFSS (Cardone *et al*, 1998) and the conserved active site pentapeptide QACGG are underlined. The arrow denotes the aspartic acid (D) residue after which the cleavage occurs during caspase-9 activation (Srinivasula *et al*, 1996). Letters coloured red represent the conserved hydrophobic residues of the ^1^CARD^97^ domain (Hofmann *et al*, 1997; Zhou *et al*, 1999). The bold and circled residues indicate the polymorphic sites where amino acid substitution takes place. Threonine (T) is exchanged for isoleucine (I), caused by the 305C>T base pair substitution. Arginine (R) is exchanged for glutamine (Q), caused by the 662A>G base-pair substitution.

Primer sequences for CASP9 and DFFA were selected by means of the DNASTAR primer select software (LASERGENE, Madison, WI, USA; data available on request). Primers for CASP9 were constructed from the nine published exon sequences ( Figure 2A

) and the mRNA sequence (GenBank accession numbers: AB019197 (exon 1); AB019198 (exon 2); AB019199 (exon 3); AB019200 (exon 4); AB019201 (exon 5); AB019202 (exon 6); AB019203 (exon 7); AB019204 (exon 8); AB019205 (exon 9); NM_001229 (mRNA)). The promoter region of the CASP9 gene could not be investigated, because the CASP9 upstream sequence was not available. Primers for DFFA exons and promoter were constructed from the sequence of an unfinished chromosome 1 clone (GenBank accession number: AL354956) and from the mRNA sequence (GeneBank accession number: NM_004401). Forward (F) and reverse (R) primers were synthesized using an ABI Applied Biosystem 392 DNA/RNA Synthesizer (Applied Biosystems) or ordered from Life Technologies (www.invitrogen.com; Life Technologies, Inc. Gaitherburg, MD, USA). Amplifications were performed according to standard procedures at our laboratory (for details see Abel *et al*, 1999) with 30 cycles of 30–45 s at 95°C, 30–45 s at 52–64°C (dependent on which exon) and 60 s at 72°C.

### DNA-sequencing

Sequencing was performed with an ABI PRISM 377 DNA Sequencer (Applied Biosystems). The sequencing reactions were made with ABI PRISM Big Dye Terminator Cycle Sequencing Ready Reaction Kit (Applied Biosystems). The PCR-products were purified with QIAquick Spin PCR purification kit (Qiagen, Hilden, Germany) or the Multiscreen PCR; 96 well purification system (Millipore Corporation, Bedford, MA, USA), and the concentrations of the PCR-products were estimated by comparison to a 100 bp-mass ladder on a 2% agarose gel. The sequence reaction products were precipitated and diluted according to standard procedures at our laboratory (for details see Abel *et al*, 1999). The samples were denatured for 3 min at 95°C on a thermo block, and loaded on a 7% acryl amide gel.

### In-silico cloning of DFFA

**Figure 3:**
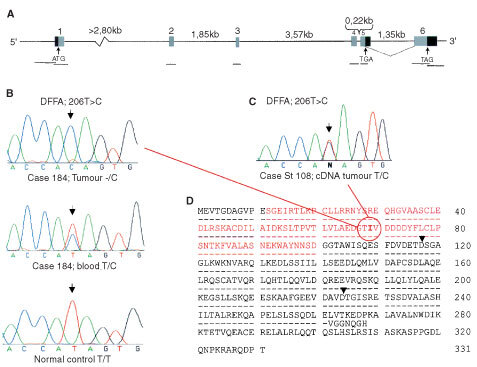
Schematic representation of the DFFA gene. (**A**) Exon-intron organization of the gene corresponding to approximately 11 kb. Exons are numbered from 1–6 in the 5′-3′ direction. Light shaded boxes represent the coding regions of each exon. Dark shaded boxes represent the 5′- and 3′-untranslated flanking regions (UTR's). The 5′ UTR is 56 bp and the most 3′ UTR is 581 bp long. The 3′ UTR of the alternatively spliced fragment after exon 5 is of unknown size. The start and stop codons are indicated by arrows. The black bars represent the eight amplified fragments (promoter-region, exon 1, 2, 3, 4, 5, 6 : 1 and 6 : 2). The dotted line represents the normal splicing of DFFA, encoding DFF45. (**B**) Rare polymorphism detected in DFFA. The 206T>C polymorphism located in DFFA exon 2. Upper panel: Amplified tumour-DNA from case 184, hemizygous -/C. Middle panel: Amplified constitutional DNA from case 184 (extracted from blood), heterozygous T/C. Lower panel: Amplified constitutional DNA from control Q170 (from blood), homozygous T/T. (**C**) Amplified tumour-cDNA from case St 108, heterozygous T/C. (**D**) The amino acid sequence of DFF45 (GenBank accession number: NP_004392). The upper sequence represents the DFF45 (331 aa) amino acid sequence, and the lower sequence represents the DFF35 (268 aa) amino acid sequence. The arrows denote the aspartic acid (D) residue after which the caspase-3 cleavage occurs (Liu *et al*, 1997; Sakahira *et al*, 1998). Letters coloured red (residues 12–100) represent the conserved N-terminal domain; CIDE (Cell-death Inducing DFF45-like Effecter; Zhou *et al*, 2001). The bold and circled residue indicates the polymorphic site where the amino acid substitution takes place. Isoleucine (I) is exchanged for threonine (T), caused by the 206T>C base pair substitution.

The genomic structure of the DFFA gene was determined by in-silico cloning. A BLAT-search of the DFFA complete coding sequence (GenBank accession number: NM_004401) was performed against ‘unfinished htgs’. One of the three hits was the unfinished chromosome 1 clone with GenBank accession number: AL354956, which was used to determine the DFFA genomic structure ( Figure 3A

and Table 2

**Table 2:**
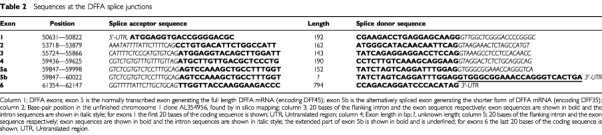
Sequences at the DFFA splice junctions

). To find a likely promoter region of DFFA we used NNPP – Eukaryotic promoter prediction by neural network available at BCM Search Launcher (url: http://searchlauncher. bcm.tmc.edu/seq-search/gene-search.html); the TSSG and the TSSW-human *Pol*II recognition programs at the same url were used to predict the most likely Polymerase II binding site.

### Expression analysis

cDNA was extracted and synthesized according to RNasy (Qiagen) and Superscript II (Promega, Madison, WI, USA) protocols. The cDNA quality was tested by PCR with GAPD-primers: sense, 5′-GGGGAGCCAAAAGGGTCATCATCT-3′, and antisense, 5′-GAGGGGCCATCCACAGTCTTCT-3′ with 30 standard cycles of amplification (30–45 s at 95°C, 30–45 s at 60°C and 60 s at 72°C). This primer pair spans two introns (intron no. 5 and 6), and generates a cDNA band at 235 bp and a genomic band at 520 bp. cDNAs showing positive PCR-results for the genomic band (520 bp) were not included in this study.

**Figure 4:**
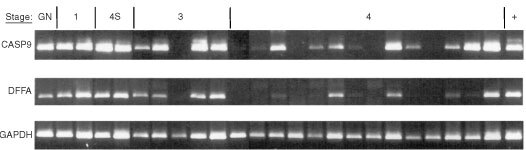
RT–PCR expression analysis of CASP9 and DFFA from all stages of neuroblastoma. GN, ganglioneuroma; 1, 2B, 3, and 4, stages of neuroblastoma; +, positive control. Upper panel: Tumour extracted cDNA amplified by RT–PCR with CASP9-primers. Middle panel: Tumour extracted cDNA amplified by RT–PCR with DFFA-primers. Lower panel: GAPDH control cDNA amplified by RT–PCR with GAPDH primers (see text). From left to right: GN, case St 151; stage 1, 118, 161; stage 4S, 125, 162; stage 3, 157, 187, St 124, St 131, St 149; stage 4, 32, 114, 126, 168, 169, 111, St 153, 95, 106, 155, 174, St 102, St 129, St 130; positive control, St 64.

The expression of CASP9, its alternatively spliced variant CASP9S, and the total expression of DFFA were tested by RT–PCR. The long (encoding DFF45) and the short alternatively spliced forms (encoding DFF35) of DFFA were not distinguished (primers are available on request). Amplifications were carried out in 50 μl and performed for 30–35 standard cycles of amplification (30–45 s at 95°C, 30–45 s at 50°C and 60 s at 72°C). The results were analyzed by running 10 μl PCR product on an ethidium bromide stained 2% agarose gel ( Figure 4

). All expression results were checked three times.

## RESULTS

### Mapping and construction of BAC-contig

According to BLAT-searches in UCSC (http://genome.ucsc.edu), CASP9 is localized to 1p36.21 and DFFA is localized to 1p36.22 very near marker D1S244 (Figure 1A). Based on the order of the seven markers according to UCSC (D1S508, GATA4H04, D1S214, GGAT3G04, D1S160, D1S503 and D1S244), the SRO of deletions that our group had previously defined (D1S508, D1S244, Ejeskar *et al*, 2001) is now localized proximal to marker D1S214 and distal to marker D1S244 (Figure 1B). Fifteen of the 16 BACs included in this study reside in this region. According to distances in the UCSC browser the SRO-region is approximately 3.5 Mb, and it partially overlaps the 500 kb homozygously deleted region defined by Ohira *et al* (2000). Four of the 16 BACs (318-F24, 513-I12, 347-P23 and 387-I22) reside in the overlapping region (Figure 1C,D). The sequence of the SP6-end from BAC 347-P23 was not determined, and could therefore not be aligned to the UCSC-contig. Both the T7-end from BAC 387-I22, and the SP6-end from BAC 318-F24 were found to reside in gap positions of the UCSC-contig. BAC 513-I12, and 347-P23 gave positive PCR-results for both DFFA and CORT. All four BACs gave positive PCR-results for marker WI-6175. Only BAC 347-P23 gave positive PCR-results for D1S244, which suggests that DFFA is localized distal to D1S244 (Figure 1C).

### CASP9 studies – detection and analysis of polymorphisms

By sequence analysis we detected four different polymorphisms in the CASP9 gene in neuroblastoma primary tumours. One was in the intron upstream of CASP9 exon 2 (IVS1-36G>A; GeneBank accession number: NM_001229) and the other three were in the exons. Of the polymorphisms in the coding region, two caused amino acid residue substitutions (305C>T and 662A>G) and the third was a silent mutation (408T>C). The 305C>T polymorphism in exon 2 caused substitution of codon 102 from a threonine to isoleucine (ACC→ATC) and we detected it as heterozygous (C/T) in three neuroblastoma patients (cases 156, St100 and St172) and as homozygous (T/T) in one patient (case 136, Figure 2B). The three patients detected to be heterozygous also displayed this polymorphism in their corresponding constitutional DNA. In addition, two of the 47 normal controls were heterozygous for this polymorphism ( Table 3

**Table 3:**
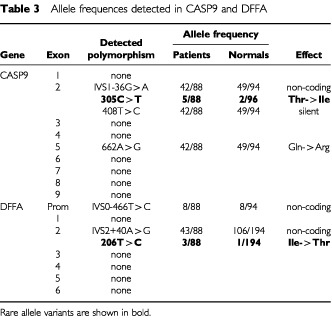
Allele frequences detected in CASP9 and DFFA

). The 662A>G polymorphism in CASP9 exon 5 caused a substitution of codon 221 from a glutamine to arginine (CAG→CGG); we found it to occur frequently.

All 44 tumours showed linkage disequilibrium for polymorphisms IVS1-36G>A, 408T>C and 662A>G. The two strict haplotypes seen were (1) A-C-G and (2) G-T-A. Of the 44 tumours, 12 were homozygous for haplotype 1; 14, of which three were informative hemizygous, were homozygous for haplotype 2; and 18 were heterozygous. Of the 48 normal controls 13 were homozygous for haplotype 1; 12 were homozygous for haplotype 2; and 23 were heterozygous (Table 3).

### CASP9 studies - expression analysis

CASP9 was expressed in all neuroblastoma stages (Figure 4). A slightly weaker expression could be seen in high-stage tumours (stage 3 and 4). CASP9 was strongly expressed in the ganglioneuroma, both tumours of stage 1, and both stage 4S tumours. Four out of five stage 3 tumours showed CASP9 expression. Ten out of 14 stage 4 tumours showed CASP9 expression; four of them (case 126, 106, St129, and St130B) showed a strong expression, while the other three were very weak. The expression of the alternatively spliced form, CASP9S, in general showed the same expression pattern as CASP9 (data not shown).

### DFFA studies – genomic structure of DFFA

The genomic structure of the DFFA gene was determined by in-silico cloning from an unfinished chromosome 1 clone (GenBank accession number: AL354956; Table 2). DFFA is organized as 6 exons (five smaller and one large) over a genomic stretch of approximately 11 kb (Figure 3A). The length of intron no. 1 could not be determined due to a gap of unknown size in the clone. No splicing of intron 5 generates the alternatively spliced form, encoding DFF35. The mRNA generated by this isoform contains normal exons 1 to 4, and a 24 bp-extended exon 5 (exon 5b, Table 2). Two different promoter sites were predicted for the forward strand of the DFFA promoter sequence (NNPP – Eucaroyotic promoter prediction by neural network; LMNL): the first between −249 to −198 from the DFFA transcription start point, and the second between −21 to +29 from the DFFA transcription start point. Use of the TSSG and TSSW-human *Pol*II recognition programs did not predict any polymerase II binding sites in the DFFA promoter sequence.

### DFFA studies – detection and analysis of polymorphisms

In DFFA, three different polymorphisms were detected by sequence analysis: one in the promoter region (IVS0-466T>C; GenBank accession number: NM 004401). One out of 44 tumours was homozygous (C/C) for the rare variant of this polymorphism, and six out of 44 were heterozygous (T/C). Eight out of 47 normal controls were heterozygous (T/C) for this polymorphism, and none could be detected that carried the rare homozygous form (C/C; Table 3). Another polymorphism, which occurred very frequently, was detected downstream of the coding region of exon 2 (IVS2+40A>G). The third was detected in the coding region of exon 2 (206 T>C) where it caused an amino acid substitution of codon 69 from a non-polar to a polar amino acid (Isoleucine to Threonine). This variant was detected in only two out of 44 tumour samples; case 184 was hemizygous -/C (Table 1; Figure 3B), and case St108 was heterozygous (T/C). RT–PCR based sequence analysis of the cDNA from case St108 revealed that both alleles (T and C) were expressed in the tumour (Figure 3C; primers are available on request). In 97 normal controls (i.e. 194 chromosomes), the C variant could only be detected in one sample as heterozygous (T/C), while 193 alleles were T (Table 3).

### DFFA studies – expression analysis

DFFA was expressed in all neuroblastoma stages (Figure 4), with a noteworthy difference between low-stage and high-stage tumours. It was expressed in the ganglioneuroma, in both stage 1 tumours, and in both stage 4S tumours. Four out of the five stage 3 tumours showed DFFA expression, but cases 157 and 187 were slightly weaker. Six out of the 14 stage 4 tumours showed DFFA expression. Four stage 4 tumours (cases 111, 106, St102 and St130B) showed a normal expression level, while the other two were very weak (cases 126, St153B).

## DISCUSSION

The heterozygous 1p-deleted SRO in neuroblastoma had already been defined both by our group (Martinsson *et al*, 1995; Ejeskar *et al*, 2001) and by others (Takeda *et al*, 1994; Caron *et al*, 1995; White *et al*, 1995; Hogarty *et al*, 2000). In the present study, we discuss an update contig of our SRO region that based on alignment searches using ‘Golden path’ at UCSC (http://genome.ucsc.edu; Figure 1B). The 500 kb homozygously deleted region on 1p36.2 in a neuroblastoma cell line (Ohira *et al*, 2000) partially overlaps our SRO (Figure 1B,C). A number of suggested but also rejected candidate tumour suppressor genes have been investigated (Maris *et al*, 1997; Martinsson *et al*, 1997; Grenet *et al*, 1998; Ejeskar *et al*, 1999, 2000). We have previously analyzed in detail one of the genes believed to reside in our SRO-region, and in the region defined by Ohira *et al* (2000), i.e. CORT (Ejeskar *et al*, 2000) without finding evidence of any role in neuroblastoma tumorigenesis (Figure 1C). In the present study we have examined two other genes in neuroblastoma primary tumours, CASP9 and DFFA. Caspase-9 has been shown to be a key effector of the apoptotic pathway in the nervous system (Hakem *et al*, 1998; Kuida *et al*, 1998) and DFF45 (encoded by DFFA) an important regulatory subunit of the DNA fragmentation factor involved in apoptosis. Both genes (CASP9 and DFFA) have been localized to 1p36.2 (Leek *et al*, 1997; Hadano *et al*, 1999; Figure 1A).

Like CORT, DFFA is believed to be localized both to our SRO-region (D1S214-D1S244) and the region defined by Ohira *et al*, 2000; Ejeskar *et al*, 2001; Figure 1C,D. However, since DFFA maps very close to marker D1S244, it was uncertain whether the gene is localized distal or proximal to this marker. PCR-assay against BACs mapped to the region of UCSC suggests that it is distal and therefore resides in our SRO-region (Figure 1C). This suggestion would accord with the proposed gene order in this region as suggested by both UCSC (http://genome.ucsc.edu) and Ohira *et al* (2000) (Figure 1D).

Forty-four neuroblastoma primary tumours were analyzed by sequence analysis for mutations in the coding region of the CASP9 and DFFA genes, and in the promoter region of DFFA. In CASP9, we found two polymorphisms causing amino acid substitutions (305C>T and 662A>G). The first was rare and caused a polar (threonine) to a non-polar (isoleucine) substitution, with only three out of 44 heterozygous and one out of 44 homozygous (T/T; see Figure 2A). The three heterozygous individuals also showed this polymorphism in their constitutional normal DNA. In the homozygous patient (case 136), the constitutional normal DNA was not available and CASP9-LOH could not be determined. This case 136 could therefore be hemizygous (-/T) for this polymorphism. We found that the 305C>T polymorphism had the same frequency (4%) in normal controls, but only in the heterozygous form.

There are several conserved regulatory motifs in caspase-9 (Figure 2C). Its large subunit contains the conserved active site pentapeptide site QACGG (Srinivasula *et al*, 1996, 1998). The ^312^PEPDA^316^-site in its amino acid sequence has been shown to be important for granzyme B cleavage (Srinivasula *et al*, 1996). Caspase-9 and Apaf-1 bind to each other via their respective NH_2_-terminal CED-3 (‘*C. elegans* Death protein homologue’) homologous domains in the presence of cytochrome *c* and dATP; they have been shown to contain a caspase recruitment domain (CARD) motif that contains several conserved hydrophobic residues (Hofmann *et al*, 1997; Zhou *et al*, 1999). Moreover, caspase-9 is negatively regulated by phosphorylation of the Ser-196 residue at the consensus motif ‘RRRFSS’ (Cardone *et al*, 1998; Figure 2C). No polymorphism found in this study could be shown to reside in any of these regulatory sites, but polymorphisms IVS-36G/A, 408T/C and 662A/G, clearly exhibited two different strict haplotypes; both were detected in approximately the same frequency in patients and normal controls, and none of the samples analyzed diverged from this pattern. Using RT–PCR, we found that caspase-9 and caspase-9S exhibited the same expression pattern, but that the expression in high stage tumours was slightly lower for both isoforms (Figure 4).

In the present study we describe the genomic organization of the DFFA gene. The gene is organized as six exons (Table 2 and Figure 3A), which is the same as that of the murine ICAD gene (Kawane *et al*, 1999). Other similarities are the alternatively spliced isoforms encoding murine ICAD-S or human DFF35, which are both generated by no splicing of intron 5. In DFFA we found one polymorphism in the promoter-region in five of 44 patients (IVS0-466T>C). One stage 3 neuroblastoma tumour (St158) was homozygous for the rare allele (C); eight of 47 normal controls displayed this polymorphism, but only in the heterozygous form (T/C). This polymorphism is located only nine bases downstream of a predicted TFIID-binding site (-TATATTTATTTAA-; as suggested by TESS-search for transcription factor binding sites).

In exon 2 of DFFA, two of 44 patients displayed one rare polymorphism in the coding region (206 T>C) that caused an amino acid substitution of codon 69 from a non-polar to a polar amino acid (Isoleucine to Threonine; Figure 3D). Case 184 was hemizygous -/C (Table 1 and Figure 3B), due to deletion, and case St108 was heterozygous (T/C). The constitutional DNA of both these two patients was heterozygous (T/C) for this polymorphism in. Only one of 97 normal controls (1%) showed this polymorphism in the heterozygous form, compared to two out of 44 of the neuroblastoma patients (4%; Table 3). Strikingly, the normal allele (T) was deleted in case 184 while only the rare C allele was retained in the tumour. However, RT–PCR based sequence analysis of the cDNA from case St108 revealed that both alleles (T and C) were expressed in this tumour (Figure 3C). DFF45 is a protein that comprises 331 amino acids (Figure 3D). In the short isoform, DFF35, the first 261 amino acids are identical. Both DFF45 and DFF35 carry two caspase-3-recognition sites (amino-acid positions 117 and 224; Sakahira *et al*, 1998; Gu *et al*, 1999). As only the full-length DFF45 functions as a chaperone for DFF40 (Sakahira *et al*, 2000), amino acids 261 to 331 seem relevant to this function (Gu *et al*, 1999). Zhou *et al* (2001) have recently reported that the N-terminal domain (NTD) of DFF45 (residues 12–100) is homologous to the NTD of DFF40. Moreover, the NTD of DFF45 is alone unstructured in solution, and its folding is induced upon binding to DFF40 NTD. Therefore, deletion of NTD from either DFF40 or DFF45 results in the production of an inactivate nuclease. Interestingly, the preserved convex hydrophobic patch of DFF40 forms van der Waal contact with the preserved concave hydrophobic patch of DFF45, through interaction between Ala-22/Tyr-75, Val-21/Val-70, and Phe-19/Ile-69 from DFF40 and DFF45, respectively (Zhou *et al*, 2001). The 206 T>C mutation/polymorphism variant causing a substitution of Ile-69, found in this study, could therefore disturb the hydrophobic contact between the NTD's. If it does, no active nucleases would be produced during apoptosis in the homozygous mutant/polymorphic cells. In the present study, we also investigated the expression of DFFA in neuroblastoma tumours (Figure 4). We selected primers of which both the normal and the alternatively spliced variant were amplified by the same PCR reaction. We detected expression of DFFA in tumours of all different stages, but found a noteworthy difference between that in low-stage and high-stage tumours. It was not or very poorly expressed in stages 3 and 4, which accords with an earlier study (Ohira *et al*, 2000).

We would conclude by suggesting that caspase-9, and, in particular, DFF45 are good candidates for the supposed tumour suppressor on 1p36.2-3. Firstly, the biology of these genes that are key players of the apoptotic signalling system renders them attractive as neuroblastoma candidate genes. Secondly, they both map to the consensus region deleted in all neuroblastoma cases with 1p deletion while DFFA also maps within the 500 kb region of homozygous loss in a neuroblastoma cell line characterized by Ohira *et al* (2000). Thirdly, we detected in two neuroblastoma cases an interesting variant polymorphism in DFFA that in one case retained as the sole allele in a tumour where the common allele had been lost by deletion. This allele variant was present only in one normal allele out of the 194 that we tested. Fourthly, low-stage tumours expressed the two genes much more distinctly than did the high-stage tumours. This was especially pronounced for DFFA: its expression in high-stage tumours was very low or absent. We would therefore suggest that, at present, neither gene be ruled out as a candidate for a neuroblastoma tumour suppressor gene. We hope that this conclusion will prompt others to study these genes, in particular DFFA, in their neuroblastoma tumour material.
